# Tape Worms

**Published:** 1860-04

**Authors:** E. C. De Puy

**Affiliations:** Freeport, Ill.


ARTICLE 5.

### TAPE WORMS.

BY E. C. DE PUY, M. D., OF FREEPORT, ILL.

Was called, October 13th, 1859, to see Mrs. S. a pale delicate woman, with a "dark, leaden appearance of the contour of the eyes," aged 29 years. The mother of two children, the youngest five years old. Found her just recovering from an attack of convulsions.

After a careful examination for the cause of the attack, I could not find anything wrong, except some decayed teeth, which were very sore, and had troubled her for some time. The functions of the various organs, digestive tube, lungs, heart, urinary and generative, were apparently healthy. As she complained of a peculiar sensation—epileptic aura—about her jaws, and running from them down the back of the neck, I was led to think the decayed teeth the cause of all her trouble. They were all extracted by her dentist, and she was put upon vegetable bitters and iron to improve her general health. Her appetite improved, and she said she felt better, only sometimes she was very nervous, and that ugly sensation about her jaws and neck troubled her some.

She remained thus until the night of the 25th of February 1860, when she had three attacks of convulsions, which were relieved by Tinct. Gelseminum, Fl. Ext. Scutellaria, Chloric Ether āā in tea-spoonful dose, and Chloric Ether by inhalation; also, friction along the spinal column, with stimulating liniment. On the following morning another careful examination was made to ascertain the cause of the convulsions. The urine was tested for albumen by nitric acid and heat, and found perfectly free—no coagulum. The bowels moved every day and appeared perfectly natural—function of the womb healthy. Ordered Hydrocyanate of Iron ʒ i., Ext. Hyoscyamus ʒ i, Mucilage q. s. to form mass, to be divided into 120 pills. Dose, one pill three times a day, to be increased according to the effect produced; also for her nervousness, Fl. Ext. Scutellaria ℥ ii. Fl. Ext. Pruni. Virginian. ℥ i Simple

Syrup ℥i. in tea-spoonful doses, as occasion required. Improving, but complains of a constant spitting of offensive mucous, insensibly rising up in her throat, which symptoms, taken in connection with the nervous phenomena, led me to suspect the presence of worms. To operate mildly upon the bowels, and partly as a vermifuge, she was ordered from 3 to 5 grain doses every four hours, of the following compound: Podophillin ʒi, Pulv. Jamaica Ginger ʒi, Sugar of Milk ℥i; mix intimately. Also to take 2 grain doses of Quinine three times a day, for its tonic effect on the nervous system. The powders operated very mildly but effectually upon the bowels, producing very offensive stools, of the consistence of tar, mixed with flattened seybala, representing the appearance of having been retained in bowels for some time; also, what we but little expected, a large number of pieces of tape worm, from a single joint to half a yard in length. The powders were continued, as it was evident that they were doing good in breaking up the habitat of, and otherwise disturbing the worm. The diet was very light—a little panada and wine.

After three or four days of such treatment, thinking that the worm was sufficiently exposed to be operated on by vermifuge or vermicide medicines, she was ordered a wine glassful of the following decoction, to be taken every half hour or every hour, as the stomach would bear, until all was taken: Bark of pomegranate root ℥ ii, boiling water Oii; steep down to one pint. To be followed, if the bowels were not much moved, or the worm not passed, by a dose of castor oil and turpentine. Commenced taking the decoction about noon; at 11 o'clock at night the husband called and said that the decoction was all taken, and the bowels had just moved, and he thought the worm had passed; and also that she was very faint, but free from pain, and otherwise comfortable, and desired to know if he should give the oil and turpentine. She was ordered a little wine and water, and rest until morning, when I visited her, and found that the bowels had just moved again, and that she was very comfortable. The last stool contained no more joints of the worm. The stool which passed at 11 o'clock contained the tape worms (Tænia Soleum) which I send you for the College Museum. The bowels have moved without purgative medicines since, and contain no more of the tape worm.

The family are "good livers." Have not eaten much pork for years. They are natives of New York State. Have been in this city some four years. The patient now (March 15th, 1860) says she feels better than she has before for years. She is very nervous sometimes, but feels nothing of that drawing of her jaws and neck.

She is now taking the Hydrocyanate of Iron pills, and Tilden's Aromatic Calasaya Wine, as a tonic and a preventive, as worms are supposed not to relish aromatic bitters. It is encouraging for the patient to know that there are fully as many "heads as tails" in the mass of worms which were passed.

